# Prediabetes: what are we talking about?

**DOI:** 10.1515/almed-2021-0034

**Published:** 2021-07-26

**Authors:** Javier Escalada San Martín

**Affiliations:** Department of Endocrinology and Nutrition, Clínica Universidad de Navarra, Pamplona, Spain; Spanish Society of Endocrinology and Nutrition, Center for Biomedical Research Network on the Physiopathology of Obesity and Nutrition (CIBEROBN), Madrid, Spain; Navarra Institute for Health Research (IdiSNA), Pamplona, Spain

Intermediate hyperglycemia (or prediabetic status) is an umbrella term for two entities: impaired fasting glucose (IFG) and glucose intolerance (GI). Different definitions have been proposed for these two overlapping conditions, according to the normal range of glycemia established. The American Diabetes Association (ADA), the World Health Organization (WHO), and the International Diabetes Federation (IDF) identify a category of hyperglycemic situations between normal glycemia and diagnosis of diabetes mellitus (DM) based on fasting glycemia in plasma or glycemia in venous plasma 2 h after 75 g OGTT (oral glucose tolerance test) [1]. More recently, it has been incorporated the possibility of diagnosis based on plasmatic HbA_1c_ levels. For IFG diagnosis, these organizations establish different ranges of fasting glycemia in plasma (100 vs. 110 mg/dL) and normal HbA_1c_ levels (5.7 vs. 6%). At present, the diagnostic criteria for prediabetes are well established (see Figure 1)

**Figure 1:**
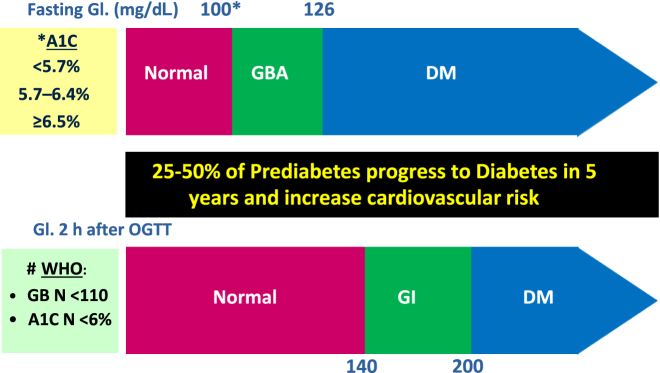
Diagnosis of preDM and DM (ADA* vs. WHO#). ADA, American Diabetes Association; DM, diabetes mellitus; IFG, impaired fasting glucose; Gl, glycemia; GI, glucose intolerance; N, normal; WHO, World Health Organization; OGTT, oral glucose tolerance test.

## Why is diagnosis of these entities important?

The prevalence of prediabetes is not a minor issue. According to the most recent data published in Spain in the *di@bet.es* study [2], the prevalence of IFG and GI is 3.4 and 9.2%, respectively. In addition, these are situations at a high risk of developing diabetes and cardiovascular disease (CV). Thus, patients with IFG are five times more likely to develop DM [3] and the cardiovascular risk is higher (nonfatal acute myocardial infarction [AMI]) and cerebrovascular events (relative risk [RR]: 1.19), with higher mortality rates (RR: 1.28), as compared to nondiabetic population [3]. On the other hand, GI is associated with a higher risk of developing diabetes, as compared to IFG. This risk is six-fold higher that in patients with normal glycemia (RR: 6.02 [95%CI: 4.66–7.38]), and up to 12 times higher in patients with both associated (RR: 12.21 [95%CI: 4.32–20.10]) [3]. GI is also associated with a higher risk of cardiovascular mortality (RR: 1.48) and general death (RR: 1.66) [3].

These data, however, is challenged by a recent cohort study [4] conducted in a sample of older adults (mean age 75.6 years) who, despite the high prevalence of prediabetes (diagnosis of IFG and/or HbA_1c_), restoration of normal glycemia, or death were more frequent than progression to diabetes. These results suggest that prediabetes may not be a solid diagnostic entity in older adults. Further research is needed in this population.

## What can we do?

A diversity of studies has been conducted to assess the effectiveness of different pharmacological and nonpharmacological interventions in the prevention of diabetes and its associated cardiovascular comorbidities in patients with prediabetes [5], [6], [7], [8], [9], [10], [11], [12], [13], [14]. The clinical benefits of primary prevention interventions for DM2 have been demonstrated in patients with GI, but not with IFG alone or in patients diagnosed with prediabetes based on HbA_1c_ levels.

Lifestyle and some antidiabetic drugs are effective in the prevention of DM. An analysis of recent studies on the effectiveness of lifestyle changes and pharmacological interventions is detailed below.

One of the latest studies on lifestyle changes is the *European Diabetes Intervention Study* [15], which is an extension of the Finnish study published in 2001 [9] and carried out in three European countries (Finland, United Kingdom and the Netherlands) to validate the findings of an initial Finnish study. After a 3.1 year follow-up, the incidence of DM decreased by 57% in the intervention group, as compared to the control group (hazard ratio [HR]: 0.42 [95%CI: 0.29–0.60], p<0.001). The incidence of DM was 65% lower in the participants who experienced a weight loss ≥ 5% 1 year after the intervention (HR: 0.35 [95%CI: 0.22–0.56], p<0.001). The incidence of diabetes further decreased in patients who maintained a constant weight loss of ≥5% for 2 and 3 years (79 and 89% reduction, respectively) [15]. It is worth mentioning an intervention study on lifestyle habits undertaken by the DE-PLAN-CAT group in Spain, which revealed a 36.5% reduction in the incidence of diabetes after an average follow-up of 4 years [16].

In relation to pharmacological interventions for the prevention of diabetes, the highest reduction in progression to diabetes was obtained in the ACT-NOW study with pioglitazone [17], which achieved a 72% reduction in the treatment group, as compared to placebo, although it was accompanied with a weight gain of 3.9 vs. 0.8 kg (p<0.001) and an increase in the incidence of peripheral edema (12.9 vs. 6.4%; p=0.007). The *Diabetes Prevention Program* (DPP) study had previously demonstrated that metformin (850 mg/BID) was effective in reducing prediabetes progression to diabetes by 31%, as compared to placebo. Of note, a 51% reduction was achieved through lifestyle changes [10]. Finally, liraglutide (a GLP-1 receptor agonist) has also been proven to significantly reduce progression to DM2 (2% in the treatment group vs. 6% in the control group) in a 3-year follow-up study [18].

In patients with morbid obesity, the most effective approach was bariatric surgery (odds ratio 0.16 [95%CI: 0.11–0.24]) [17].

Conclusive data on its effect on cardiovascular morbidity and mortality has not been obtained due to the short duration of studies. Acarbose has demonstrated to be effective in reducing cardiovascular complications in the STOP-NIDDM study [12], but this finding is based on only 48 events, and its results should be interpreted with caution since assessing its effects on CV morbidity and mortality was not within the scope of this study.

A recent meta-analysis revealed that the use of lifestyle changes and medication in adult patients with prediabetes reduced the incidence of DM2 by 39 and 36%, respectively. Nevertheless, medication is less effective in the long-term (its effects are not maintained over time), whereas the effects of lifestyle changes are maintained after the intervention [19].

This study confirms previous results, as weight loss was found to be the primary factor associated with progression to DM2. The risk of progression to DM2 decreased by 7% per lost kilogram which, in clinical terms, is very relevant.

## The accusation of “prediabetes business”

There is solid evidence about the beneficial effects of lifestyle interventions. However, the main limitation of these interventions is patient’s difficulty in maintaining the recommended lifestyle changes; therefore, pharmacological prevention emerges as an alternative for some patients. Despite the data available on the effectiveness of pharmacological interventions in reducing the incidence of diabetes, the indication of hypoglycemic drugs for prediabetes has not yet been approved.

Some authors suggest that prediabetes is a diagnostic invention intended to make the population use medications unnecessarily [20]. This statement, however, is challenged by solid data. On the one hand, only ADA [21] clearly considers the possibility of using metformin in the prevention of DM2 in patients at a very high risk of developing diabetes, such as patients with GI (level of evidence A), IFG (E level), or HbA_1c_ of 5.7–6.4% (E level), especially in the presence of a BMI >35 kg/m^2^, age < 60 years (higher efficacy within this range of age demonstrated in the DPP study) or in women with a previous diagnosis of gestational diabetes (A level of evidence). On the other hand, a study recently published in *Diabetes Care* revealed that the use of metformin in patients with prediabetes is <1%, which demonstrates that pharmacological interventions are not frequent in the prevention of DM2 [22].

Finally, after doubts were raised about the reality or relevance of prediabetes, research is increasingly focused on it as a real entity and its categorization into subtypes [23]. The different metabolic groups identified are associated with future complications associated with prediabetes, insulin resistance, future risk of DM2 and mortality. These subphenotypes might reflect key pathological features potentially associated with metabolic complications in the future. However, the purpose of phenotype identification is not to classify individual patients in clinical practice, but to provide guidance about the design of strategies for the prevention and management of cardiovascular and kidney diseases and DM2. We expect further news about this interesting condition to better understand prediabetes.
